# Human Geophagia, Calabash Chalk and Undongo: Mineral Element Nutritional Implications

**DOI:** 10.1371/journal.pone.0053304

**Published:** 2013-01-07

**Authors:** Peter W. Abrahams, Theo C. Davies, Abiye O. Solomon, Amanda J. Trow, Joanna Wragg

**Affiliations:** 1 Institute of Geography and Earth Sciences, Aberystwyth University, Ceredigion, United Kingdom; 2 Department of Geology and Mining, University of Jos, Jos, Nigeria; 3 British Geological Survey, Keyworth, Nottingham, United Kingdom; Indiana University, United States of America

## Abstract

The prime aim of our work is to report and comment on the bioaccessible concentrations – i.e., the soluble content of chemical elements in the gastrointestinal environment that is available for absorption – of a number of essential mineral nutrients and potentially harmful elements (PHEs) associated with the deliberate ingestion of African geophagical materials, namely Calabash chalk and Undongo. The pseudo-total concentrations of 13 mineral nutrients/PHEs were quantified following a nitric-perchloric acid digestion of nine different Calabash chalk samples, and bioaccessible contents of eight of these chemical elements were determined in simulated saliva/gastric and intestinal solutions obtained via use of the Fed ORganic Estimation human Simulation Test (FOREhST) *in vitro* procedure. The Calabash chalk pseudo-total content of the chemical elements is often below what may be regarded as average for soils/shales, and no concentration is excessively high. The *in vitro* leachate solutions had concentrations that were often lower than those of the blanks used in our experimental procedure, indicative of effective adsorption: lead, a PHE about which concern has been previously raised in connection with the consumption of Calabash chalk, was one such chemical element where this was evident. However, some concentrations in the leachate solutions are suggestive that Calabash chalk can be a source of chemical elements to humans in bioaccessible form, although generally the materials appear to be only a modest supplier: this applies even to iron, a mineral nutrient that has often been linked to the benefits of geophagia in previous academic literature. Our investigations indicate that at the reported rates of ingestion, Calabash chalk on the whole is not an important source of mineral nutrients or PHEs to humans. Similarly, although Undongo contains elevated pseudo-total concentrations of chromium and nickel, this soil is not a significant source to humans for any of the bioaccessible elements investigated.

## Introduction

Humans ingest soil both deliberately – a practice known as geophagia or geophagy – and accidentally, with consequent implications to their mineral nutrition [1]. Thus following the encounter with digestive fluids, chemical elements can be solubilised from soils and are potentially available for absorption, the so-called bioaccessible soil content. For example, geophagical soils consumed by ethnic Bengali communities in the UK were found by Abrahams et al. [2] to be a significant source of bioaccessible iron (Fe). Since this chemical element is an important mineral micronutrient with Fe deficiency being widespread throughout the world [3], the consumption may be of benefit to the geophagist although with the quantities of soil that can be deliberately consumed (e.g., up to c. 65 g/day [2]; 8–108 g/day with a median of 28 g/day [4]; 2.5–219 g/day with a median of 41.5 g/day [5]) so-called Guidance Levels [6] could be exceeded. Furthermore, Abrahams et al. [2] highlighted the risk of soil-lead (Pb) toxicity affecting pregnant women – a group of human society who are especially associated with geophagia – and their foetus. Conversely, the absorption of elements into the human body following soil consumption can also be reduced attributable to, for example, the adsorptive properties of ingested earth materials that can lower bioaccessible concentrations. Hooda et al. [7] indicated the sorption potential of some geophagical soils in lowering the bioaccessibility of copper (Cu), Fe and zinc (Zn), although other materials were identified to be a source of calcium (Ca), magnesium (Mg) and manganese (Mn) that humans could potentially utilise.

A review of the literature clearly indicates that geophagia is not limited to any particular age group, race, sex, geographic region or time period, though today the practice is most obviously common amongst the world’s poorer or more tribally-oriented people and is, therefore, particularly extensive in the tropics [8]. A number of accounts relating to geophagia in Nigeria can be found in the literature [9]–[13], and here the practice is noted to be especially associated with pregnant women who consume earth materials to alleviate the symptoms of morning sickness. Calabash chalk – also known (according to language/locality) as Argile, Calabar stone, Calabash clay, Ebumba, La Craie, Mabele, Ndom, Nzu, Poto and Ulo – is a generic term used for naming these Nigerian geophagical materials.

The migration of people from societies where geophagia is especially prevalent results in a cultural transfer of the practice to countries that many would consider to be not typically associated with this deliberate consumption. Thus, in the UK, geophagia is known to be associated with immigrants from south Asia [2], [14]–[15] and west Africa [16]–[17], with the latter consuming Calabash chalk that has been imported from Nigeria and sold in ethnic shops. In some developed countries, concern has been expressed about this consumption – not only in the UK [18], but also in Canada [19] and the USA [20] – because of the Pb content. The UK Food Standards Agency [21] have reported (presumably total) Pb concentrations in Calabash chalk that range from 8.2 mg/kg to 16.1 mg/kg, whilst Dean et al. [16] determined a mean total content of ≈ 40 mg/kg. While these total concentrations are significantly greater than previous World Health Organisation guideline limits of 1 mg Pb/kg in foodstuffs, an important consideration is the bioavailability (defined here as the fraction that reaches the human systemic circulation from the gastrointestinal [GI] tract) of soil-Pb. The bioavailability of this Pb – and other chemical elements – is strongly dependant on bioaccessibility since if an element is not bioaccessible it will not be available for absorption [22], and both bioavailability/bioaccessibility are influenced by a number of soil variables (mineralogy, particle size and morphology) as well as factors associated with the human individual, such as age, sex, genetics and socioeconomic status [1], [23]. However, being dependant on *in vivo* studies either on humans [24] or (more commonly) human surrogates such as pigs [25] and rats [26], the bioavailability of soil chemical elements is more difficult and involved to evaluate. Consequently, much use has been made of *in vitro* bioaccessibility (IVBA) tests that mimic the conditions of the human GI environment and determine the bioaccessibility of ingested soil chemical elements. Initially relatively simple experimentation was undertaken, using reagents such as hydrochloric acid to simulate the conditions of the human stomach (e.g., [5], [27]–[28]), but with the recognition that such procedures ignore the changes in the Eh/pH regime and kinetics during passage of soil through the GI system, increasingly more sophisticated IVBA tests have been developed [22], [29]–[30]. However, problems are evident with the use of these various IVBA procedures - there is a lack of Certified Reference Materials (CRMs) that are needed for the evaluation of the accuracy of the analysis, there is insufficient *in vivo* information against which the bioaccessible concentrations can be compared, and the various models employed produce different results – though the BioAccessibility Research Group of Europe (BARGE) has recently (and after we undertook the experimental work described below) developed and published information about a fasted state IVBA method that begins to address some of these issues [31] and which has been correlated against *in vivo* data for arsenic (As), cadmium (Cd) and Pb [32].

Despite advances made in the development of IVBA procedures, there has been only a limited application regarding their use on geophagical materials. Indeed, some recent studies on deliberately consumed earth materials can be criticised either because of their continued use of simplified IVBA procedures, or their reliance on total chemical element determinations [33]–[34]. The main aim of our work is to report and comment on the bioaccessible concentrations of a number of elements found in commercially available Calabash chalk materials purchased from markets in Nigeria. To determine these concentrations, we subjected the Calabash chalk samples to an IVBA test that was originally developed for assessing the bioaccessibility of soil organic pollutants when the geophagists are in a fed-state (we use this fed procedure because the main consumers of Calabash chalk are pregnant women who ingest these earth materials either just before or after mealtimes). Coincidentally, as this research was being undertaken, a commercial geophagical sample – known as Undongo, the Swahili word for soil - from Kenya was made available to us. With current interest being evident regarding human geophagia in Africa, we also included this soil in the IVBA experimentation, and provide here the bioaccessible data derived from this material.

## Materials and Methods

### 2.1 Sample Details and Collection

Nine varieties of Calabash chalk, selected on the basis of their obvious differences in appearance (such as colour, lamination and shape), were purchased from markets located in Jos (Plateau State, Nigeria) and Zaria (Kaduna State). Seventeen market vendors and 526 women who were in hospital or were attending antenatal clinics, were questioned about the origins and use of Calabash chalk in Nigeria. It is the intention to publish the findings of this survey at a later date, but we briefly report some pertinent information here. Pregnant women from the Igbo tribe – a large ethnic group of eastern Nigeria – were recorded as the main users, and comments were made on the effectiveness of the ingested soils/rocks in limiting vomiting during pregnancy and reducing over-salivation: there is no suggestion that the Calabash chalk is being consumed to aid mineral nutrient supplementation. The daily amount consumed varies, but generally is c. 5–10 g. However, differences are apparent between pregnant and non-pregnant women: the former tend to ingest more (up to 20 g/day) with consumption occurring either before or after mealtimes to prevent vomiting, whereas the latter consume soil generally when in a fasted state. For both groups of women, consumption occurs by gnawing chunks of the geophagical material.

**Figure 1:**
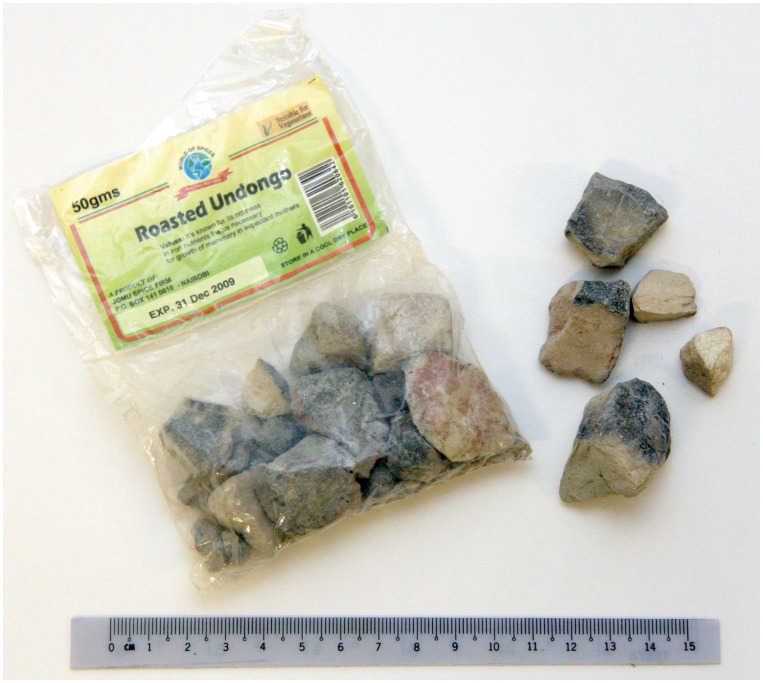
Roasted Undongo purchased from a small supermarket in Kenya. The labelling, not fully distinct on the image, states a “richness of iron” that is of benefit to pregnant women and their unborn child. Photograph: Peter W. Abrahams.

The Undongo sample was obtained from a small supermarket in the Ukambani hills, Kenya. This material is purchased in labelled polythene bags (Figure 1) that contain blocky units of ‘roasted’ soil some 50 g in total. The labelling highlights the richness of Fe in the soil and its value to pregnant women and their foetus, although no details about how the material should be consumed (e.g., in a fasted or fed state; how much should be ingested/day) is provided.

### 2.2 Sample Preparation

Samples were oven-dried at 40°C for 48 hrs prior to disaggregation using a porcelain mortar and pestle and sieving through a 2000 µm aperture nylon mesh. Subsamples of the <2000 µm fine earth fraction were then: (I) further sieved through a 250 µm aperture nylon mesh and, (II) ground and sieved through a 150 µm aperture nylon mesh. The <2000 µm, <250 µm and <150 µm subsamples were retained for subsequent analysis.

### 2.3 Determination of Pseudo-total Concentrations

The <150 µm subsamples were subjected to a nitric-perchloric acid digestion following the procedure described by Thompson and Wood [35]. These authors note the effectiveness of this acid mixture in decomposing clay minerals and a number of primary minerals, though some soil constituents (if present) are not fully digested, and consequently the method is often regarded as a procedure that determines, in association with appropriate instrumentation, pseudo-total (i.e., an approximation) rather than true total concentrations. However, our use of fine (i.e., <150 µm) material in this experimental procedure facilitates sample decomposition and optimises the release of chemical elements into solution. Analysis of the sample solutions was undertaken using inductively coupled plasma mass spectrometry (ICP-MS) for six soil trace elements (Cd, cobalt [Co], chromium [Cr], Cu, nickel [Ni] and Pb), whilst another trace element, Zn, and a number of major (Ca, Fe, potassium [K], Mg) and minor (Mn, sodium [Na]) elements were determined by atomic absorption spectrometry.

### 2.4 Determination of Bioaccessible Concentrations

For our assessment of the bioaccessible concentrations, we used the Fed ORganic Estimation human Simulation Test (FOREhST) procedure. This method was initially developed to assess the bioaccessibility of soil polycyclic aromatic hydrocarbons (PAHs, widespread organic pollutants) under simulated fed conditions. Most IVBA procedures are concerned with inorganic soil constituents, and simulate fasted conditions that – relative to the fed state – are associated with lower pH conditions, so providing the most conservative estimate of bioaccessibility [36]. However, since pregnant women who consume Calabash chalk ingest the material either just before or after mealtimes, we used the FOREhST method to quantify soil bioaccessibility in a fed-state where the geophagist is consuming food. To investigate the impact of ingested foodstuffs on bioaccessibility *per se*, we also subjected the soil samples to the FOREhST procedure digestion pH and transit conditions but did not add the foodstuff component of the experimental method. To summarise, soil chemical element bioaccessibility was: (I) determined using the FOREhST method, a fed-state experimental procedure that is appropriate to use since the main consumers in this investigation are pregnant women who ingest the geophagical materials just before or after mealtimes, and (II) assessed using the FOREhST method where the food component of the *in vitro* procedure is omitted, but where other experimental variables (transit times, solution pH, enzyme concentrations) are the same as (I), so enabling the effect of food alone on bioaccessibility to be evaluated.

The FOREhST procedure is a IVBA test, carried out at 37°C to simulate human body temperature, and utilising end-over-end rotation that replicates the churning of soil and fluid in the gut. The stages involved in the methodology represent the saliva/gastric and intestinal (i.e., duodenal and bile) phases of the human GI system. Briefly (for a detailed description, see Cave et al. [36]), 0.3 g of each soil/shale sample was weighed into an individual extraction bottle to which was placed 4.5 ml of simulated saliva solution (pH = 6.8±0.5). After 5 min, 9 ml of simulated gastric solution (pH = 1.4±0.5) was added to produce a mixed saliva/gastric solution phase that had a final pH of 1.6±0.2. Following 2 hr of rotation in a water bath calibrated to 37°C, sample solutions were retrieved from each extraction bottle, and retained for chemical element quantification. This procedure was then repeated on another set of weighed soil/shale samples but, following the 2 hr of rotation with the saliva/gastric solution, 9 ml of simulated duodenal (pH = 8.1±0.2) solution and 4.5 ml of bile fluid (pH = 8.2±0.2) was then added to each extraction bottle, producing a final solution that had a pH of 6.0±0.5. The extraction bottles were rotated in the water bath for a further 2 hrs after which the sample solutions were retrieved for chemical analysis. This version of the methodology leads to leachate sample solutions being collected both at the end of what we term the saliva/gastric and intestinal phases of extraction. Chemical element quantification of these solutions was undertaken using ICP-MS instrumentation. Because of analytical considerations (e.g., a variety of Na-containing reagents are used in the FOREhST procedure, with deleterious implications for the determination of bioaccessible Na), we restrict our focus to the bioaccessible concentrations of eight elements (Co, Cr, Cu, Fe, Mn, Ni, Pb and Zn): these bioaccessible concentrations associated with the geophagical materials are reported in units of mg/kg.

Since the bioaccessibility of inorganic soil constituents is dependent upon, amongst other variables, particle size, the FOREhST procedure was applied to sieved materials of <2000 µm and <250 µm. We chose these particle sizes firstly because the geophagists are ingesting the bulk material (which explains our use of the <2000 µm fraction), and secondly because IVBA methods undertaken for human health risk assessments typically use <250 µm particles. Soil chemical element bioaccessibility also depends on whether foodstuffs have been ingested and, as previously mentioned, we subject the geophagical materials to (I) a scenario of fed-state (F-S) conditions where a freeze-dried oatmeal and rice porridge infant food supplemented with sunflower oil is used as the foodstuff in the experimental procedure as described by Cave et al [36], and (II) simulated fed GI pH and transit conditions but where no food has been consumed by the geophagist (i.e., no rice porridge/sunflower oil is used in the laboratory method: a fed-state, no food [F-SNF] scenario that enables the effect of food alone on bioaccessibility to be evaluated).

To summarise the IVBA methodology, eight solutions were obtained for ICP-MS chemical element quantification from each geophagical sample subjected to the FOREhST procedure. These solutions represent the saliva/gastric and intestinal phases of extraction on <2000 µm and <250 µm particle sizes, and simulated F-S and F-SNF conditions.

### 2.5 Determination of other Sample Variables

To provide some background information about the geophagical materials, a number of procedures were undertaken in the laboratory. A 1∶2.5 w/v distilled water suspension was used for pH determination undertaken on the <2000 µm geophagical samples, whilst the method used to assess the cation exchange capacity (CEC) was based on that of Bascomb [37]. Organic carbon (OC) content was quantified from <150 µm material following a modified version of the procedure described by Walkley and Black [38] whereby the OC is oxidised with an acid dichromate solution. Soil colour was measured by comparison with a colour chart [39].

### 2.6 Analytical Quality Control (AQC) Procedures

Trow [40] details the appropriate AQC procedures that were undertaken to measure the robustness of our analytical data. For example, two CRMs were included in the analysis to assess the accuracy of the determined pseudo-total concentrations. Replicates of these CRMs and two of the geophagical samples allowed the calculation of precision, whilst detection limits (calculated as 3 x standard deviation of mg/l blank values multiplied by appropriate dilution factor) were determined from the results derived from the analysis of blank samples.

A CRM was included when undertaking the FOREhST procedure and one geophagical sample was randomly chosen for replication. Blank samples were also included and, where appropriate, the bioaccessible concentrations of the *in vitro* solutions associated with the geophagical materials were ‘blank deducted’.

### 2.7 Calculation of Maximum Absorption Potential (MAP) Values

To aid the evaluation of the importance of the geophagical materials in supplying chemical elements to humans we have calculated MAP values for Co, Cr, Fe, Mn and Ni. Such calculations are based on the following assumptions:

- That the amount of soil/shale material consumed by the geophagist is 20 g/day.
- Since mineral elements are mainly absorbed from the small intestine of the GI tract, the highest concentration determined from the leachate solutions associated with this phase of the *in vitro* procedure – irrespective of particle size or F-S/F-SNF simulated conditions – was used for the calculations. For Cr, where all the leachate concentrations were below the detection limit, the latter threshold was used in the calculation to determine a <MAP value.
- That all of the chemical elements solubilised in the simulated intestinal phase of the *in vitro* procedure are absorbed by the geophagist. We recognise that this is actually very unlikely [41]–[42], and as such the MAP values are an overestimate of the bioavailability of the chemical elements investigated in this way. Nevertheless, we justify use of the MAP values, since they provide a worst-case scenario that allows us to assess the implications of the bioaccessible concentrations determined from the *in vitro* leachate solutions.

## Results

### 3.1 Results from the Employed AQC Procedures

Table 1 provides a summary of the AQC results associated with the determination of the pseudo-total concentrations. Some problems in the quality of the data are evident – e.g., raised concentrations of Ca in the blank samples, the poor accuracy of Na determined from the two CRMs – but the results following the analysis of the geophagical materials are discussed in light of the insight that the AQC procedures have provided.

**Table 1:** Analytical accuracy, precision and detection limits determined from samples (CRMs; blanks) subjected to a nitric-perchloric acid digestion.

		Certified Reference Material		Detection limit (mg/kg)^c^
		GBW07407^a^	SGR-1^b^	
Ca	Accuracy^d^:Precision^e^:	-f-f	812.9	552
Cd	Accuracy:Precision:	19723	1374	0.1
Co	Accuracy:Precision:	10211	918	0.3
Cr	Accuracy:Precision:	6911	949	3.5
Cu	Accuracy:Precision:	10210	1099	4.1
Fe	Accuracy:Precision:	830.6	883.3	1794
K	Accuracy:Precision:	712.3	221.1	40
Mg	Accuracy:Precision:	302.6	1370.6	306
Mn	Accuracy:Precision:	728	1098.4	0.4
Na	Accuracy:Precision:	415	191.5	26
Ni	Accuracy:Precision:	13911	1368	12
Pb	Accuracy:Precision:	9413	12219	0.6
Zn	Accuracy:Precision:	9423	10013	4.0

a laterite soil produced by the National Research Centre for Certified Materials, China;

b Green River shale produced by U.S. Geological Survey;

c calculated as 3 x standard deviation of mg/l blank values multiplied by appropriate dilution factor;

d defined here as the deviation of the measured observation from the true (certified) value, and calculated as (mean of five measurements/certified) x 100%;

e precision = coefficient of variation (%) determined from five replicates of one sample;

f cannot be calculated since below detection limits.

Relative to the pseudo-total contents, the bioaccessible concentrations determined from the leachate solutions following the *in vitro* FOREhST procedure are more difficult to interpret from the perspective of AQC. Many concentrations are close to or below detection limits – thus, for example, having implications for quantifying the precision of our analysis determined from replicate samples – whilst CRMs that can be used to evaluate accuracy are very limited for this type of analysis. The British Geological Survey (BGS) reference soil no: 102 has a bioaccessible guidance value – determined using the Unified BARGE Method (UBM) IVBA procedure [31] – of 13±6 mg Pb/kg for the stomach phase (note this method simulates unfed conditions). This CRM was used in our analysis, but the two blank sample solutions associated with the F-SNF saliva/gastric phase – the closest simulated conditions that we employed compared to the UBM method – of our experimental procedure were found to have notably higher Pb concentrations than the BGS reference material solutions (indeed the solutions of all the geophagical samples associated with this F-SNF phase had a Pb content that was less than those of the blank solutions). We conclude that the CRM soil, and all the geophagical materials, are adsorbing Pb and consequently lowering the simulated saliva/gastric solution concentrations of the F-SNF scenario (a reaction that, as we highlight later, is also apparent for some of the other determined elements, most notably Cu and Zn). The UBM procedure is run at a stomach pH of 1.2, lower than the FOREhST method we employed where the pH following the saliva/gastric phase of the F-SNF scenario is typically 1.6±0.2. The latter pH range was observed for the solutions of the geophagical samples following the saliva/gastric F-SNF extraction in our work, but notably the pH of the BGS CRM soil solutions following this phase was 4.5–4.8. The increased pH will be a significant factor in accounting for the solution concentrations of Pb observed in our analysis of this CRM, with the implication being that we cannot assess the accuracy of the Pb concentrations determined in our work because of the significant pH differences compared to the UBM procedure from which the bioaccessible guideline value of 13±6 mg Pb/kg is obtained.

Eight samples were replicated when undertaking the IVBA procedure in order to determine the precision of this analysis. However, because a number of solution concentrations were below the limits of detection, the reproducibility of analysis can only be quantified from a more restricted number of duplicated samples: Table 2 provides a summary of the precision estimates determined in our study.

**Table 2:** Precision of analysis quantified from sample replicates subjected to the *in vitro* FOREhST procedure.

	Co	Cr	Fe	Mn	Ni
Median precision^a^	10.2	9.4	20.8	5.5	6.7
*n* b	4	2	6	4	3

a precision = coefficient of variation (i.e., standard deviation/mean x 100%) determined from replicated samples;

b number of replicated samples from which median precision calculated.

### 3.2 Some General Observations Relating to the Geophagical Materials

**Figure 2:**
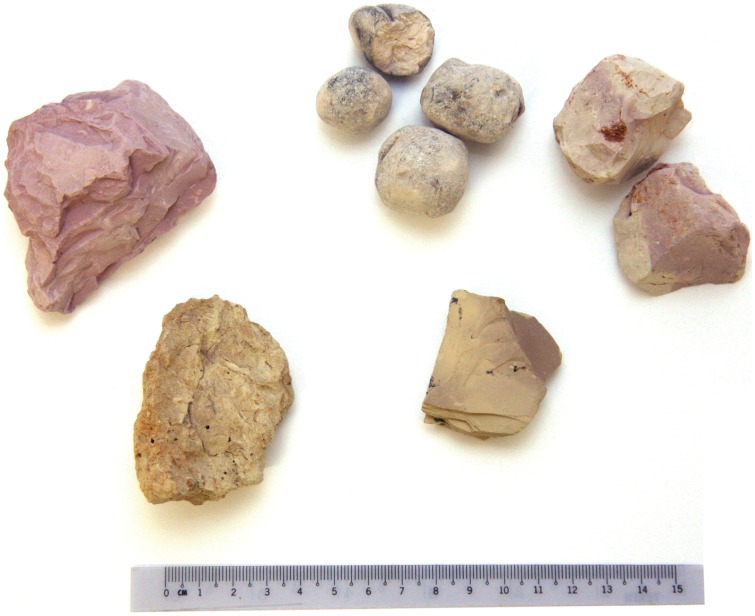
Five of the Calabash chalk samples investigated in our study. These materials are shales (top left [sample 8 as listed in Table 3] and bottom right [sample 4]) and dried/baked soils (samples 9, 6 and 5, top middle and right, and bottom left, respectively). Photograph: Peter W. Abrahams.

Most of the Calabash chalk samples are clay-rich soil materials that have been dried and/or baked into blocky or spherical units, though some are laminated shales (i.e., argillaceous sedimentary rocks; Figure 2). Table 3 records some details relating to these materials. All of the Calabash chalk samples have a very low OC content ranging from <0.1–0.4% (median  = 0.1%) suggesting that the geophagical soils have been excavated from sub-surface horizons rather than (more organic enriched) topsoils. These inorganic geophagical materials have a CEC rating that we interpret as mainly varying from very low (i.e., <6 cmol_c_/kg) to medium (i.e., within the range 12–25 cmol_c_/kg), and all samples of Calabash chalk are acidic in reaction (minimum-maximum pH = 3.4–6.4; median = 5.1). In contrast, Undongo has a high CEC (32 cmol_c_/kg) and an alkaline reaction (pH = 7.7), though like the Calabash chalk it is associated with a very low OC content (0.1%).

**Table 3:** Place of purchase, general area of origin, consumers and appearance of the geophagical materials.

Sample	Purchase location	Origin	Consumers^a^	Appearance^b^
1	Jos Main Market	Jos Plateau, central Nigeria	PW, N-PW, C	Clay blocky units: 10YR 7/3 very pale brown
2	Jos Main Market	Jos, central Nigeria	PW, N-PW, C	Clay blocky units: 10YR 7/3 very pale brown
3	Jos New Market	Southeast Nigeria	PW, N-PW	Spherical clay units: 5YR 7/2 pinkish grey
4	Jos Main Market	Southeast Nigeria	PW, N-PW	Shale: 10YR 7/2 light grey and 2.5Y N4/0 dark grey
5	Jos Main Market	Southeast Nigeria	PW, N-PW	Shale: 2.5Y 7/2 light grey and 2.5Y 7/4 pale yellow
6	Jos Main Market	Southeast Nigeria	PW, N-PW	Clay blocky units: 5R 6/2 pale red and 5YR 7/1 light grey
7	Jos New Market	Southeast Nigeria	PW, N-PW, C, M	Clay blocky units: 5Y 6/2 pale red and 5YR 8/1 white
8	Jos Terminus Market	Jos Plateau, central Nigeria	PW, N-PW	Laminated clay blocky units: 5R 6/2 pale red
9	Zaria Main Market	Imo State, Southeast Nigeria	–	Spherical clay units: 5R 6/4 pale red and 5YR 7/2 pinkish grey exterior; 5YR 8/1 white interior

a PW = pregnant women; N-PW = non-pregnant women; C = children; M = men;

b includes Munsell soil colour [39].

### 3.3 Pseudo-total Concentrations

A summary of the pseudo-total concentrations determined from the samples of Calabash chalk is provided in Table 4. The concentrations of Pb approximate or slightly exceed typical ‘average’ values that are associated with shales and present-day soils [43]–[44], and the contents are similar to Calabash chalk analysis that has been previously reported by Dean et al. [16]. However, a notable feature of the majority of the results in Table 4 is that while they are mostly within the normal range of total soil/shale concentrations, for most of the elements – Ca, Cd, Co, Cr, Cu, Fe, K, Mg, Mn, Na, Ni and Zn – the contents in many samples are below what may be regarded as an average value (in coming to this conclusion, it needs to be appreciated that normal/typical total concentrations of soil chemical elements are mostly reported from topsoils – that the Calabash chalk soils appear not to be – which are likely to be enriched because of natural processes such as adsorption by surface organic matter or low-level anthropogenic pollution). The pseudo-total concentrations of some pieces of Calabash chalk are an exception to this general observation with, for example, two samples – both shales – containing the highest amounts of Ca (1769 mg/kg and 2473 mg/kg, respectively), Co (both 17 mg/kg), Fe (37070 mg/kg and 46670 mg/kg) and Mg (12936 mg/kg and 21176 mg/kg) recorded in our study. However, no concentration of any chemical element in any sample of Calabash chalk can be regarded as excessively high. In contrast, the Undongo sample contains elevated pseudo-total concentrations of Cr and Ni (152 mg/kg and 126 mg/kg, respectively) compared to many soil materials, and is distinct from the Calabash chalk in having a low Pb and K content (8 mg/kg and 210 mg/kg, respectively).

**Table 4:** Descriptive statistics summarising the pseudo-total content (mg/kg) of the Calabash chalk samples, and concentrations determined from the Undongo sample.

	Ca	Cd	Co	Cr	Cu	Fe	K
Calabash chalk^a^:							
Median	<552	0.1	2.5	23	6.9	18186	1978
Min. – Max.	<552–2473	0.1–0.7	2.0–17	8.0–67	<4.1–18	6498–46670	830–3354
IQR	–	0	6.5	31	8.8	20348	1036
MBIV (%)	–	–	130	67.4	63.8	55.9	26.2
Undongo:	<552	0.1	9.1	152	18	7758	210
	**Mg**	**Mn**	**Na**	**Ni**	**Pb**	**Zn**	
Calabash chalk:							
Median	507	18	235	27	37	23	
Min. – Max.	<306–21176	<0.4–572	107–514	23–49	20–43	11–87	
IQR	–	273.8	407	26	23	76	
MBIV (%)	–	760.6	86.6	48.1	31.1	165.2	
Undongo:	1426	57	3914	126	8	19	

a *n* = 9. IQR = inter-quartile range; MBIV = median-based index of variability (calculated as quartile deviation/median x 100%, where the quartile deviation is half the inter-quartile range). Some IQR and MBIV values cannot be calculated.

### 3.4 Bioaccessible Concentrations

For both the saliva/gastric and intestinal phases of the F-SNF scenario, all the *in vitro* leachate solutions have Pb concentrations less than the sample blanks, suggesting an adsorption of this PHE by soil/shale constituents (furthermore, for the intestinal phase, any Pb not adsorbed by the geophagical material is likely to be precipitated/complexed by the increased pH and enzyme concentration [45]–[46]). The blanks associated with the F-S scenario of our *in vitro* experimentation have a notably lower Pb concentration when compared with the blank solutions of the F-SNF scenario. These results are indicative of adsorption by the food component of the experimental procedure, a reaction exacerbated by the pH of the blank solutions (the blank solutions associated with the saliva/gastric part of the F-S scenario [median pH = 4.4] are notably less acidic than those of the F-SNF [pH = 1.8] phase). Binding of Pb by some of the geophagical materials is also apparent from the F-S *in vitro* results (i.e., the Pb content of the geophagical solutions are less than those determined from the blanks), especially by particles <250 µm in size. However, some materials are also a source of Pb since associated leachate solution concentrations are greater than those of the blanks, though these solution concentrations are close to or below the limits of detection (which are 0.25 mg/kg and 0.7 mg/kg for the gastric/stomach and intestinal phases of the F-S scenario, respectively). A maximum concentration of 0.65 mg Pb/kg (sample 1, <2000 µm soil) and 2.5 mg Pb/kg (sample 9, <2000 µm soil) was recorded from the F-S saliva/gastric and intestinal phases, respectively.

The results associated with the Cu and Zn bioaccessible concentrations are similar to those observed for Pb. The blank solution Cu contents associated with the F-SNF scenario are elevated (22 and 8 ng/ml for the saliva/gastric and intestinal phases, respectively), and many leachate solutions contain less than these concentrations and so are indicative of adsorption by the geophagical materials. Some intestinal leachate solutions contain more Cu than the F-SNF blanks, but at concentrations that can not be detected with confidence. Similar conclusions apply to the Cu concentrations of solutions associated with the F-S scenario, with only one sample (number 4) yielding more (1.1 mg/kg linked with the saliva/gastric phase) than can be robustly detected. The majority of the Zn concentrations obtained from the F-SNF scenario are below the content found in the blanks (8.1 and 10.1 ng/ml for the saliva/gastric and intestinal phases, respectively), though nearly all of the <2000 µm geophagical materials associated with the F-SNF saliva/gastric phase yield leachate solution concentrations slightly above the blank concentrations (but none are greater than the robust limit of detection which is 5.3 mg/kg). For the F-S scenario, again the majority of the leachate solution Zn concentrations are either below the blank solution contents or below the limits of detection (the latter are 1.2 mg/kg and 2.4 mg/kg for the saliva/gastric and intestinal phases of this scenario, respectively). However, one F-S leachate solution (Undongo, <250 µm soil, saliva/gastric phase) yielded a detectable concentration of 4.7 mg Zn/kg.

For the remaining chemical elements (i.e., Co, Cr, Fe, Mn and Ni) considered in the bioaccessibility study, whilst a number of leachate solutions have concentrations below those of the blank solutions, many are above so indicating the potential of ingested soils/shales to be sources of these mineral elements that humans can subsequently absorb. Tables 5 and 6 provide a summary of these bioaccessible concentrations, with a number being reported as below the limits of detection.

**Table 5:** Bioaccessible concentrations (mg/kg) associated with the F-SNF saliva/gastric and intestinal phases of the *in vitro* procedure.

	Saliva/gastric leachate phase									
	<250 µm					<2000 µm				
	Co	Cr	Fe	Mn	Ni	Co	Cr	Fe	Mn	Ni
Calabash chalk:										
Median	0.70	0.28	25	6.3	+^a^	+	0.25	39	3.0	+
Min. – Max.	<0.14–3.5	0.16–0.40	<14–116	<1.8–253	<0.80–4.5	<0.14–1.8	0.14–0.44	16–54	<1.8–136.5	<0.80–2.4
*n* b	8 of 9	2 of 9	5 of 9	8 of 9	8 of 9	8 of 8	8 of 9	4 of 8	7 of 8	8 of 8
										
Undongo:	1.5	1.7	<blk^c^	9.6	<0.80	3.3	2.3	<blk	11.9	<0.80
	**Intestinal leachate phase**									
	**<250 µm**					**<2000 µm**				
	**Co**	**Cr**	**Fe**	**Mn**	**Ni**	**Co**	**Cr**	**Fe**	**Mn**	**Ni**
Calabash chalk:										
Median	<0.70	<2.8	<28	5.9	<blk	<0.70	<2.8	74	4.8	<1.7
Min. – Max.	<0.70–3.1	All <2.8	<28–158	<3.6–187	All<blk	<0.70–1.5	All <2.8	<28–53	<3.6–83	<1.7–3.2
*n* b	9 of 9	9 of 9	7 of 9	7 of 9	0 of 9	8 of 8	4 of 8	2 of 8	5 of 8	6 of 8
Undongo:	1.6	<2.8	<28	<3.6	<blk	<0.70	<2.8	<blk	<3.6	0.36

a cannot be computed since value is at the interface of detectable/not detectable concentrations;

b number of samples with concentrations above those of the sample blanks out of the total number of Calabash chalk samples analysed;

c <blk = less than sample blank concentrations.

**Table 6:** Bioaccessible concentrations (mg/kg) associated with the F-S saliva/gastric and intestinal phases of the *in vitro* procedure.

	Saliva/gastric leachate phase									
	<250 µm					<2000 µm				
	Co	Cr	Fe	Mn	Ni	Co	Cr	Fe	Mn	Ni
Calabash chalk:										
Median	0.25	<0.17	16	<4.8	<2.5	+^a^	<0.17	25	<4.8	<2.5
Min. – Max.	<0.12–0.56	All <0.17	13–56	<4.8–32	All <2.5	<0.12–0.48	<0.17–0.24	18–63	<4.8–28	All <2.5
*n* b	8 of 9	4 of 9	4 of 9	8 of 9	7 of 9	8 of 8	8 of 8	8 of 8	7 of 8	6 of 8
Undongo:	0.30	<0.17	<9.6	<4.8	<2.5	0.20	<0.17	<blk^c^	<4.8	<2.5
	**Intestinal leachate phase**									
	**<250 µm**					**<2000 µm**				
	**Co**	**Cr**	**Fe**	**Mn**	**Ni**	**Co**	**Cr**	**Fe**	**Mn**	**Ni**
Calabash chalk:										
Median	+	<2.0	15	<3.9	<1.1	<0.12	<2.0	13	<3.9	<1.1
Min. – Max.	<0.12–0.40	All <2.0	<6.1–9.5	<3.9–23	All <1.1	<0.12–0.31	All <2.0	8.8–24	<3.9–15	All <1.1
*n* b	9 of 9	8 of 9	9 of 9	9 of 9	8 of 9	8 of 8	4 of 8	4 of 8	6 of 8	6 of 8
Undongo:	0.30	<2.0	10	<3.9	<1.1	0.12	<2.0	35	<3.9	<1.1

a cannot be computed since value is at the interface of detectable/not detectable concentrations;

b number of samples with concentrations above those of the sample blanks out of the total number of Calabash chalk samples analysed;

c <blk = less than sample blank concentrations.

## Discussion

A common concern that is expressed about geophagia is that ingested earth materials are potentially a source of PHEs that can have a clinical or sub-clinical toxic effect on an individual (e.g., Shellshear et al [47]; Wedeen et al. [48]). With exceptions (e.g., the Cr and Ni content of the Undongo sample), the geophagical materials examined in our study are not enriched in PHEs – such as Pb – when considering the pseudo-total concentrations and comparing them to those of other soil/shale materials. Nevertheless, because Calabash chalk contains pseudo-total concentrations of Pb that are well in excess of those found in the majority of foodstuffs (which are typically well below 1 mg/kg [49]–[50]) some organisations within developed countries such as the UK have expressed anxieties about its consumption and are trying to restrict its importation and use. In any evaluation of the chemistry of geophagical materials however, it is the bioaccessible concentrations that are more important than the total contents. For Cu, Pb and Zn many of the leachate solutions are either less than the blanks, suggestive of adsorption by the geophagical materials, or have concentrations that cannot be robustly quantified. For Pb, the maximum concentration found in our study was only 2.5 mg/kg associated with the intestinal phase of the F-S procedure. The instinct is to conclude that these materials are generally not an important source of this mineral element, or Cu and Zn, but there is evidence for Pb that there is no apparent safety threshold with a human health risk associated with even low-level exposures [51].

A number of leachate solutions have Co, Cr, Fe, Mn and Ni concentrations lower than the blanks that are again suggestive of the adsorptive properties of the geophagical materials. For those leachate concentrations that are greater than the blank solutions, a number cannot be quantified with confidence making interpretation of the data difficult. Should ingested soils release bioaccessible mineral elements, it would generally be expected that finer particles will be a more significant source due to their greater effective surface area, that the acidic gastric environment will promote the release of many elements relative to the more alkaline intestinal part of the human digestion system, and that the solubility within the GI tract is greater in F-SNF rather than F-S scenarios. The limited fully quantifiable data of this study make it difficult to confirm such generalisations, but we can make some observations about the importance of these ingested geophagical materials regarding their role in supplying Co, Cr, Fe, Mn and Ni to consumers of such products. Table 7 details the MAP values of these five chemical elements by humans. These values are compared against Reference Nutrient Intake (RNI) values (defined as the amount of a nutrient that is adequate for nearly all – i.e., 97.5% - of a population group; [41]) for adolescent females aged 15–18 years and women of child-bearing age (i.e., the main consumers of Calabash chalk as identified in our questionnaire survey). For varying reasons, no RNI is proposed for Co, Cr, Mn and Ni, but the MAP observed for Fe is ≈ 21.4% of the RNI for this chemical element. The potential of ingested Calabash chalk being a significant source of this essential mineral nutrient to geophagists is thus demonstrated, and is similar in magnitude to that previously identified by Abrahams et al. [2], but it needs to be noted: (I) that this observation is based on the highest concentration of Fe detected in the intestinal leachate solutions, and (II) that 100% absorption of the bioaccessible Fe is extremely unlikely [42], [52]. The median detectable concentrations of Fe in the intestinal leachate solutions indicate that the average amount absorbed following a 20 g/day ingestion of Calabash chalk – again assuming that all the soluble Fe is incorporated into the human body – is <0.56 mg/day, a value that is <3.8% of the RNI indicated in Table 7. Our conclusion is that generally Calabash chalk is not a significant source of Fe to the geophagist. A similar conclusion can also be made for the Undongo sample. Despite the “richness of iron” displayed on the packaging of this product, Undongo has neither the (pseudo) total nor bioaccessible concentrations to justify this statement: a maximum concentration of 35 mg/kg recorded from the intestinal phase of the *in vitro* leachate procedure equates to a MAP of just 0.7 mg/day (assuming a soil intake of 20 g/day, and 100% absorption of this soluble Fe), some 4.7% of the RNI displayed in Table 7.

**Table 7:** MAP values of five mineral elements following the consumption of 20 g of Calabash chalk by human geophagists, and a comparison with: (I) RNI values for adolescent 15–18 year old females and women of child-bearing age, and (II) SULs/GLs for a 60 kg adult.

	Co	Cr	Fe	Mn	Ni
MAP (mg/day)^a^	0.06	<0.06^b^	3.16	3.74	0.06
RNI^c^	No RNI^d^	No RNI	14.8	No RNI^e^	No RNI^f^
SUL/GL^g^	1.4	10^h^	17^i^	4^j^	No SUL/GL

a Calculated using the highest concentration recorded from the solutions associated with the intestinal phase of the *in vitro* leachate procedure employed in our study, and assuming that all of the element released into solution is absorbed by the geophagist;

b since all intestinal Cr concentrations are below detection limits, this value is derived using the highest such threshold value determined in our study;

c values (mg/day) from UK Department of Health (DoH, [41]);

d no RNI in this form can be given since although an essential element, Co is utilised by humans only as a constituent of vitamin B_12_ that is obtained from the consumption of meat, supplements/pharmaceuticals or fortified foods;

e human Mn deficiency has not been observed outside experimental studies and since intakes thus appear adequate the DoH [41] set no RNI for this chemical element;

f No RNI established since Ni deficiency has not been observed in humans and their requirement for this metal is unknown (but could be as low as 5 µg/day [55]);

g values expressed as mg/day [6];

h GL applies to trivalent Cr (the naturally occurring valency state of this chemical element that is found in soils);

i for guidance purposes, a supplemental intake of 17 mg/day would not be expected to produce adverse effects in the majority of people. This is based on data referring to the ferrous form of Fe;

j GL for supplemental intake.

We are not the only researchers who have concluded that ingested soils are not a significant source of Fe to the geophagist. The *in vitro* work of Hooda et al [7] indicated how the sorption potential of some geophagical soils can lead to a reduction of Fe concentrations in the simulated GI fluids. These findings, however, contradict those from other research. Thus, following the use of an IVBA procedure that simulated unfed conditions, both Abrahams et al. [2] and Smith et al. [53] highlighted the potential of ingested soils in supplying a significant amount of Fe to the geophagist. Other research (e.g., [5], [27]–[28]) has also suggested this, though a criticism that can be directed to these investigations is that the laboratory methodology is too simplistic compared to the human GI environment.

The importance of ingested Calabash chalk as a potentially deleterious source of Co, Cr, Fe, Mn or Ni can be evaluated by making reference to the Safe Upper Levels (SULs) or Guidance Levels (GLs) outlined in Table 7. Determined by the UK Expert Group on Vitamins and Minerals (EVM, [6]), SULs represent an intake that can be consumed daily over a lifetime – and note, many geophagists do not deliberately consume earth materials throughout their life, but instead partake only during certain periods such as pregnancy. Our questionnaire survey undertaken in Nigeria indicates that the pregnant women commence geophagia at the onset of vomiting/perceived over-salivation about two months into their pregnancy, ceasing the practise some 4–6 months later – without significant health risk, while GLs (which are based on more limited data) give an approximate indication of levels that would not be expected to cause adverse effects. Previous research [2] has shown that geophagists can potentially exceed the GL for Fe, but the rate of ingestion of Calabash chalk and its bioaccessible Fe content would seem to pose little threat to humans consuming such materials: the MAP of 3.16 mg/day noted in Table 7 is 18.6% of the GL associated with this chemical element. Of the five chemical elements detailed in Table 7, it is the MAP of Mn (3.74 mg/day) that is the nearest to the threshold levels provided by the EVM (who consider that a supplemental intake of up to 4 mg Mn/day in addition to the diet would be unlikely to produce adverse effects).

As previously highlighted, the Undongo contains elevated pseudo-total concentrations of Cr and Ni. However, regarding this soil material, the MAP of Cr is <0.056 mg/day, well below the GL of 10 mg Cr/day (Table 7). The EVM [6] could not establish a SUL or GL for the supplemental intake of Ni, but noted that dietary intakes of this metal can cause flare-ups of dermatitis since it is a potent skin sensitizer. Certain foodstuffs such as soya beans typically contain elevated concentrations of Ni, and a supplementary diet comprised of such constituents – that provided an oral intake of 0.49 mg/day – was found to trigger symptoms of hand eczema in Ni-sensitive female patients [54]. Assuming a 20 g/day ingestion of Undongo however, the bioaccessible concentrations of Ni – from which a MAP of <0.02 mg/day can be calculated – do not appear to be a threat to the geophagist.

### Conclusions

Any ingested geophagical material has the potential to release mineral nutrients and PHEs when they come in contact with digestive fluids. However, our investigations following the use of two scenario’s associated with the FOREhST IVBA procedure, indicate that at rates of consumption of 20 g/day, the Calabash chalk materials on the whole are not a significant source of mineral nutrients or PHEs to humans (this finding applies also to Undongo despite the Fe enrichment stated on the labelling of the product). Indeed, with many *in vitro* leachate solutions containing concentrations of, for example, Pb that are less than the blanks employed in our analysis, the geophagical materials are capable of adsorbing chemical elements, so preventing their absorption. Our results further suggest that foodstuffs can be a sink for Pb in the GI environment. Whilst the reported concerns about ingested Calabash chalk may be lessened with such findings we would still advocate caution about the use of such materials: their microbial sterility, for example, is questionable and needs to be investigated.
